# Fetal heart rate changes and labor neuraxial analgesia: a machine learning approach

**DOI:** 10.1186/s12884-023-05632-3

**Published:** 2023-05-22

**Authors:** Efrain Riveros-Perez, Javier Jose Polania-Gutierrez, Bibiana Avella-Molano

**Affiliations:** Department of Anesthesiology and Perioperative Medicine, Medical College of Georgia, Augusta University, 1120 15th street BI-2144, Augusta, GA 30912 USA

**Keywords:** Fetal heart rate, Anesthesia and Analgesia, Machine learning

## Abstract

**Background:**

Neuraxial labor analgesia has been associated with fetal heart rate changes. Fetal bradycardia is multifactorial, and predicting it poses a significant challenge to clinicians. Machine learning algorithms may assist the clinician to predict fetal bradycardia and identify predictors associated with its presentation.

**Methods:**

A retrospective analysis of 1077 healthy laboring parturients receiving neuraxial analgesia was conducted. We compared a principal components regression model with tree-based random forest, ridge regression, multiple regression, a general additive model, and elastic net in terms of prediction accuracy and interpretability for inference purposes.

**Results:**

Multiple regression identified combined spinal-epidural (CSE) (p = 0.02), interaction between CSE and dose of phenylephrine (p < 0.0001), decelerations (p < 0.001), and the total dose of bupivacaine (p = 0.03) as associated with decrease in fetal heart rate. Random forest exhibited good predictive accuracy (mean standard error of 0.92).

**Conclusion:**

Use of CSE, presence of decelerations, total dose of bupivacaine, and total dose of vasopressors after CSE are associated with decreases in fetal heart rate in healthy parturients during labor. Prediction of changes in fetal heart rate can be approached with a tree-based random forest model with good accuracy with important variables that are key for the prediction, such as CSE, BMI, duration of stage 1 of labor, and dose of bupivacaine.

**Supplementary Information:**

The online version contains supplementary material available at 10.1186/s12884-023-05632-3.

## Background

Labor combined spinal-epidural (CSE) is associated with rapid onset of analgesia [1]. CSE is associated with increased risk of fetal bradycardia in the absence of maternal hypotension [2]. Although a multivariate analysis revealed that only pain scores and maternal age were independent predictors of fetal bradycardia after neuraxial blockade, there is still controversy regarding the contribution of CSE to this outcome [3]. The mechanism underlying the altered fetal heart rate after CSE with opioids remains elusive; however, uterine hypertonicity secondary to a rapid drop in plasma catecholamine levels may be the culprit [4]. The effect of neuraxial medications on fetal heart rate is poorly understood. It is unknown whether any differences in risk exist between fentanyl and bupivacaine when used in CSE procedures. There are reports of uterine hyperactivity and fetal bradycardia following subarachnoid administration of fentanyl during labor [5].

Given the multifactorial nature of changes in fetal heart rate during labor, our study aims to perform statistical inference and prediction of changes in fetal heart rate during active labor in healthy pregnant women by comparing three different machine learning methods: multiple linear regression, splines, and elastic lasso. We aim to identify the contribution of different predictive factors to changes in fetal heart rate in the context of normal labor and create an accurate model to predict those changes in future parturients scheduled for vaginal delivery.

## Methods

This study has been reported according to the STROBE guidelines [6]. According to the Declaration of Helsinki was registered at https://clinicaltrials.gov/, under the registration code NCT05399979. After approval by the Augusta University Committee A Review Board (Protocol #: 1,567,908. 03/16/2021), a retrospective chart review was conducted for healthy parturients admitted for vaginal delivery between November 2017 and August 2020. In agreement with the American Society of Anesthesiologists (ASA), we defined healthy pregnant patients as an ASA Physical Status Classification system II [7]. Information for the study was obtained from the institutional electronic information system of Augusta University Medical Center. Patients older than 18 years old with healthy term pregnancies (> 37 weeks) were included. Patients with baseline systolic blood pressure < 90 and diastolic blood pressure < 60, maternal fever, and uterine tachysystole > 5 contractions in 10 min before neuraxial block were excluded. Also, patients with American College of Obstetricians and Gynecologists (ACOG) category III fetal heart rate tracing were excluded. After neuraxial block, a wedge was placed under the patient and adequate hydration was ensured as preventive measures. Lateral positioning was exclusively used in patients with non-reassuring tracing, as it may provoke sensory block asymmetry.Prenatal and maternal variables were obtained from the institutional health.

documentation system. Variables recorded included age, weight, height, body mass index (BMI), race, number of pregnancies and prior deliveries, gestational age, cervical dilatation, oxytocin use, neuraxial block type used, neuraxial medications employed, fentanyl, morphine, and bupivacaine dose used in the initial bolus, hemodynamic variables, vasopressors use, and fetal heart rate variables. The dataset was de-identified after the data collection.

### Regression methods

We compared a principal components regression model with tree-based random forest, ridge regression, multiple regression, a general additive model, and elastic net in terms of prediction accuracy and interpretability for inference purposes. Accuracy performance was evaluated with mean square error (MSE). Model coding and performance estimation, and general statistical analysis are provided as part of the R packages available from the Comprehensive R Archive Network (https://cran.r-project.org/web/packages/). The specific packages employed are referenced below in the corresponding section for each statistical learning method.

### Principal components regression

Principal components regression (PCR) is a dimensionality reduction method that uses the technique of principal components analysis (PCA). In the context of a linear relationship between predictors and response, reducing dimensionality leads to a reduction in variance at a relatively low cost of bias for the statistical learning method used. PCR is a linear approximation that uses new coordinates (principal components) to contain most of the information present in the predictors. The new principal components are then used to fit a least-squares linear model [8, 9]. A more detailed description of the method and mathematical background is found in the supplemental material 1. The R “pls” package was used to fit the PCR model (https://cran.r-project.org/web/packages/pls/index.html).

### Random forest

The random forest algorithm is a supervised learning method used for regression and classification problems. It breaks the feature space into small fractions to grow a randomized tree predictor on each piece of data and then aggregates those predictors together [10, 11]. The method entails bootstrap aggregation (bagging) to build regression trees. Since every new sample created by bootstrap uses all predictors, it would be expected for those predictors to be correlated. The random forest algorithm involves random selection of a limited number of predictors every time a new decision tree is created, reducing the tree correlation. The result is a robust method with good prediction accuracy. The mathematical foundations of this method can be found in the supplemental material 1. The R package “randomForest” was used to build the forest in this study.

### Ridge regression

Linear models are easy to interpret and simple to understand; however, they may be associated with overfitting depending on sample size and data distribution. To mitigate this risk, different methodologies aim at reducing variance without a concomitant increase in bias to improve model prediction accuracy. One of those techniques is ridge regression. Ridge regression uses shrinkage of linear regression coefficients without getting to the point of selecting variables [12, 13]. In many problems where data are not obtained from experimental design, non-orthogonality of predictor variables makes it impossible to assign proper weight to the individual features, thereby limiting the predictive accuracy of a linear model. Ridge regression employs a penalty function governed by a tuning parameter that affects the loss function of residual sum of squares of linear regression. As the tuning parameter increases, the model becomes less flexible, leading to lower variance and higher bias. The ideal value for the tuning parameter optimizes the bias-variance trade-off. The mathematical foundations of this method can be found in the supplemental material 1. The R package “glmnet” was used to fit ridge regression in this study.

### Multiple regression

We used multiple linear regression to detect relationships between multiple predictors (categorical and continuous) on the fetal heart rate decrease and their relative importance. Backward variable selection was performed to identify independent variables. For deleting variables, the F ratio criterion was 4.0, which is the squared value of a t-test for the hypothesis that the coefficient of the predictor in question equals zero. A p-value less than 0.05 was considered statistically significant.

### General additive model

General additive models (GAM) extend the linear regression models to include non-linear relationships between some or all features and the response [12, 13]. A non-linear function is applied and is different for each feature. Although non-linearity is part of the function, the interpretation is still part of the linear framework. The functions are nonparametric in the sense that the shape of predictor functions is determined by data directly and not by a small set of parameters. This allows for prediction without knowing the patterns beforehand. The function can be expressed in terms of coefficients as weights and a basis expansion. The basis expansion introduces non-linearity into each predictor-response relationship and corresponds to the general term of spline. Splines are smooth functions that can be understood as polynomials that cover a small range. The number of splines is a parameter that needs to be defined when this model is used. The parameter λ penalizes the splines. As λ value increases, the spline gets smoother until it becomes a straight line. The optimal value for λ is determined by cross-validation.

### Elastic net

Elastic net regression is a regularization method that uses the penalization of lasso and ridge regression on the loss function of ordinary least squares (OLS) regression [14, 15]. Elastic net improves the lasso limitations. It incorporates a quadratic component to the penalty function (the ridge regression component) to make the lasso constraint more convex. The procedure to find the elastic net coefficient estimates starts with the calculation of the ridge regression coefficients followed by shrinkage of those coefficients using the lasso algorithm. Elastic net performs well when there are highly correlated independent variables. During the shrinkage procedure, the L1 norm of the lasso selects variables. On the other hand, the L2 norm of ridge regression makes the L1 component of the penalty more stable.

## Results

### Patient characteristics

Our database consisted of 1077 patients admitted to the labor and delivery unit. A total of 39 predictors including demographic (age, race, and anthropometric measures), obstetric (gravidity, parity, weeks of gestation, and contractions), anesthetic (type of neuraxial block and medications), and hemodynamic (maternal blood pressure, heart rate, and vasopressors need) variables were collected for each patient. The demographic variables are reported as mean +/- SD and frequency (%) where appropriate (Table 1). The incidence of fetal bradycardia and prolonged deceleration was 56 cases per 1000 parturients per year. The response variable was the percentage decrease in fetal heart rate after administration of neuraxial labor analgesia. The dataset was randomly split into training and test sets.

**Table 1:** Demographic Characteristics

Variables	Mean +/- SD	Frequency (%)
Age	27 +/- 6.2	-
Weight	87.1 +/- 20.2	-
Height	162.9 +/- 7.7	-
BMI	32.8 +/- 7.7	-
Race		
African american	-	542 (50.2%)
White	-	420 (38.9%)
Hispanic	-	82 (7.6%)
Asian	-	28 (2.6%)
Indian	-	6 (0.5%)
CSE	-	472 (43.8%)
Latent phase of labor (< 4 cm of dilation)	-	344 (31.9%)

CI, confidence interval. Mean (SD) for continuous variables and frequency (%) for categorical variables. **BMI**, Body mass index. **CSE**, combined spinal-epidural

### Model fitting and error

We started by fitting a multiple regression model, where we identified a statistically significant association between percentage decrease in fetal heart rate and presence of decelerations before the neuraxial block (p < 0.001), vasopressor dose required to stabilize blood pressure (p < 0.001), performance of combined spinal-epidural (p = 0.015), and intrathecal bupivacaine dose (p = 0.03). In order to improve the interpretability of the model, we limited the number of predictors and were able to observe two findings. First, CSE (p = 0.02), interaction between CSE and phenylephrine dose (p < 0.0001), decelerations (p < 0.001), and the total bupivacaine dose (p = 0.03) affect the response variable in a statistically significant manner. Second, the adjusted R-square is low (0.08), indicating significant variability in the data. This latter finding can be explained by additional predictors not included in the original model and the lack of linearity in the association between predictors and response. Multiple regression, in this case, is a model that helps us make statistical inferences about the association, but its predictive precision is poor. For this reason, we explored other statistical learning methods for prediction purposes.

**Fig. 1:**
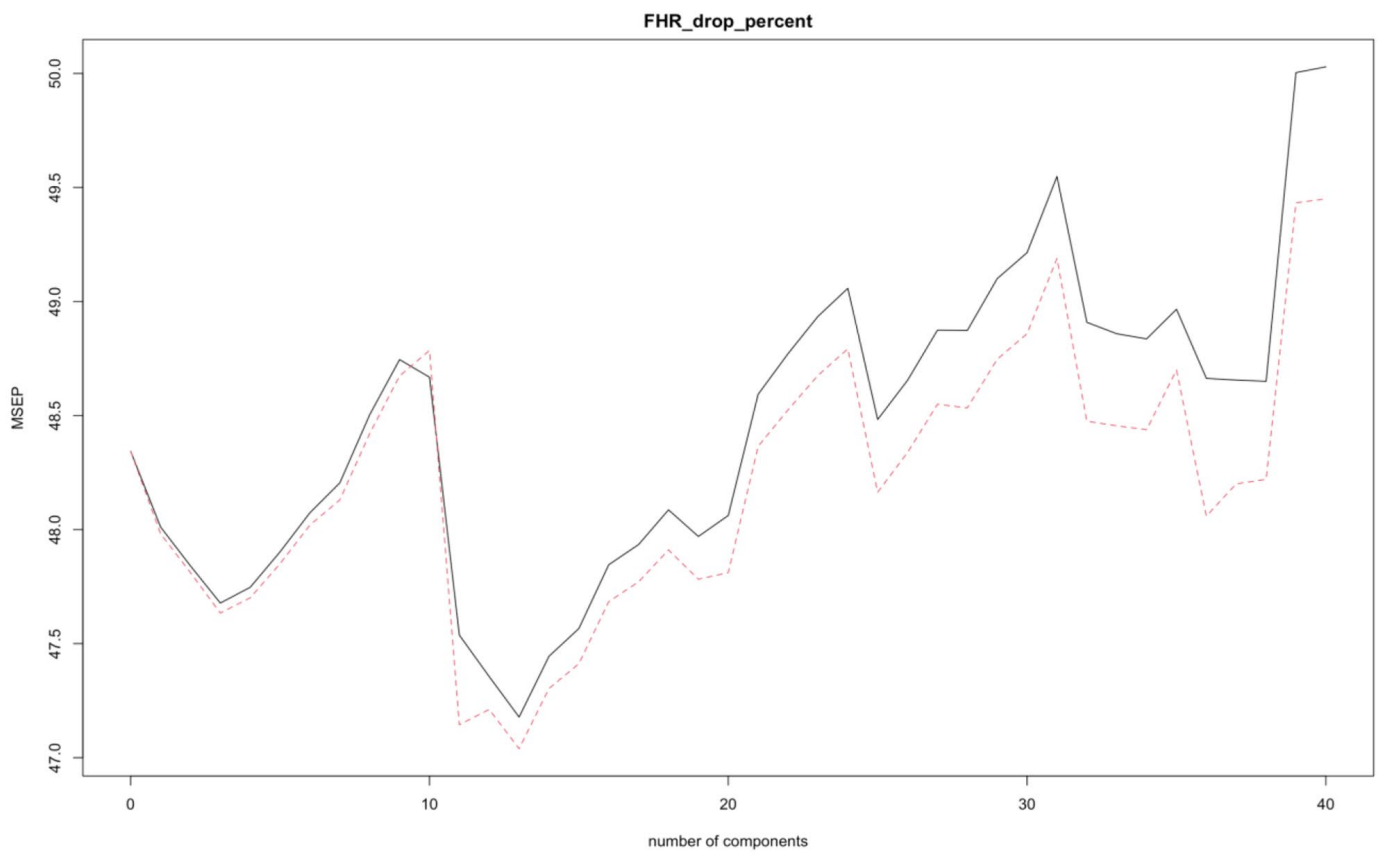
Mean square error (MSE) as a function of the number of components. Minimum MSE with 13 principal components

We performed a PCR analysis after scaling the variables. The first two principal components account for 98.5% of the variance (information contained in the data). MSE defined as the average of residual sum of squares, is minimized when we use 13 principal components (MSE = 42.93) (Fig. 1).

**Fig. 2:**
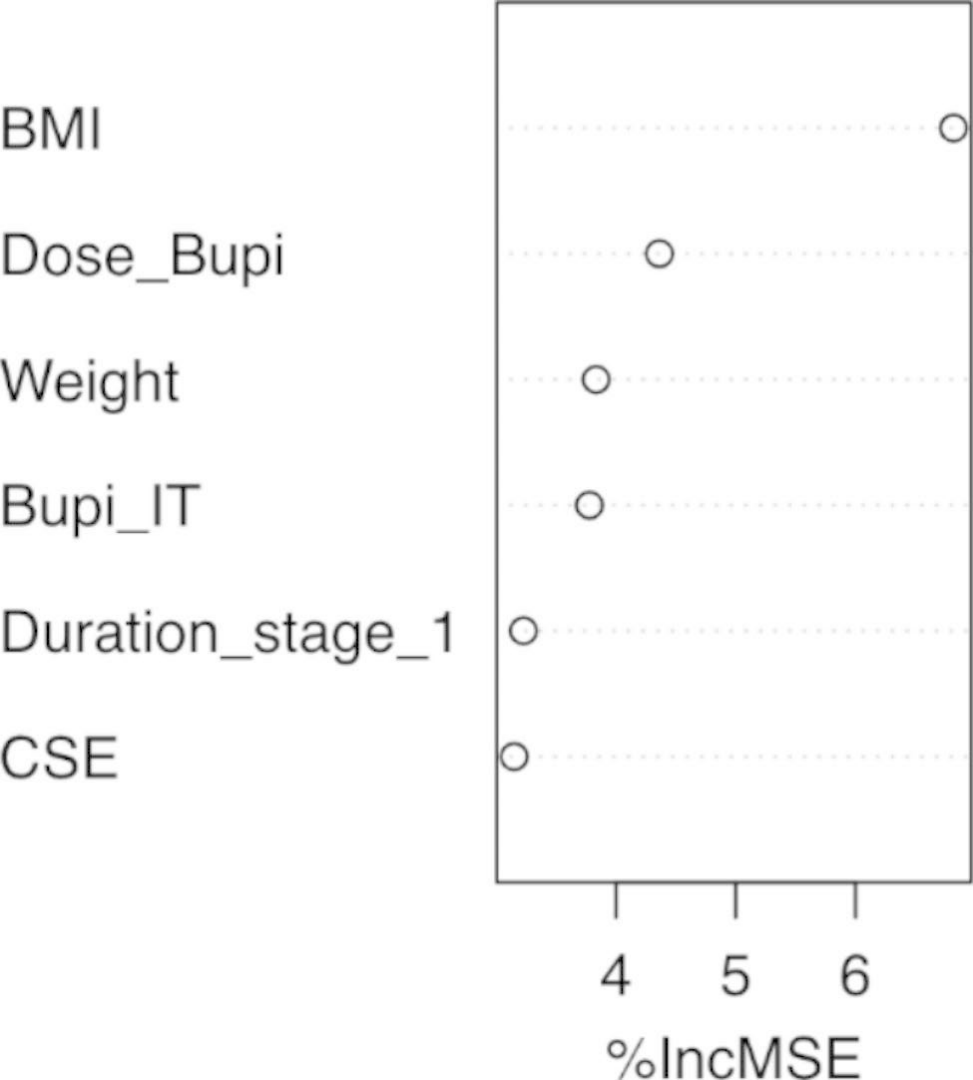
Variable importance. Random forest model. %IncMSE, percentage contribution to mean square error reduction. BMI, Body mass index. CSE, Combined spinal and epidural. Bupi, Bupivacaine. IT, intrathecal

Random forest algorithm with 13 predictors randomized per split showed MSE of 0.92. The variables identified in order of importance based on MSE and node purity were duration of stage 1 of labor, BMI, CSE use, and intrathecal bupivacaine dose (Fig. 2). Random forest exhibited a significantly better predictive accuracy than PCR and identified two predictors that were statistically significant with multiple regression (CSE and bupivacaine dose).

Ridge regression using a penalty function showed a λ parameter of 15.79 associated with the minimum MSE found by cross-validation. The minimum MSE was 42.08, comparable to the one found by PCR. The general additive model using splines for the continuous variables showed a value for MSE of 42.05, whereas the elastic net that combines the penalty function of ridge and the lasso showed MSE of 43.1.

## Discussion

Changes in fetal heart rate during labor after administration of neuraxial analgesia represent a complex and multifactorial challenge [3]. Severe drops in fetal heart rate are associated with adverse neonatal outcomes [16, 17]. Fetal bradycardia has been observed after neuraxial labor analgesia via a mechanism involving uterine hypertonus mediated by catecholamine levels [18, 19]. Isolation of individual causative factors for the development of changes in fetal heart rate after labor analgesia has been elusive, and identification of those patients at risk for fetal bradycardia continues to represent a challenge to clinicians [3, 4]. The introduction of artificial intelligence algorithms to solve medical problems is a novel addition to the clinical decision-making process. The statistical learning methods presented here provide valuable insight into the multiple factors associated with changes in fetal heart rate. These methods may also help predict decreases in fetal heart rate after neuraxial labor analgesia in clinical practice.

The multiple regression model showed the relationship between several predictors and the response variable. Although the role of the model to explain the response variable is poor (low adjusted-R2), the statistical significance for the coefficients of CSE, total bupivacaine dose, decelerations, and the total phenylephrine dose in the presence of CSE demonstrates that there is a definite trend for such features. Adjusted-R2 represents the scatter around the regression line, and its low value in our study highlights the significant variability between individuals and the possible contribution of factors not included in the model. Considering that fetal bradycardia may occur in 1.1% of normal labor [20], confirmatory analysis of our findings using a larger sample would be required to recommend using our multiple regression model as a predictive tool [21]. Although prolonged decelerations may be an incidental finding without clinical repercussions, it may also denote a fetus at increased risk of developing fetal distress [22]. We may argue that the predictors cited above could help identify patients who may develop fetal bradycardia during labor and monitor these patients closely for fetal distress signs.

Combined spinal-epidural has been associated with fetal bradycardia [22]. Cheng et al. found that the degree of sympathetic block and pain relief are risk factors for fetal bradycardia after CSE [23]. Yang et al. proposed that fetal bradycardia after CSE occurs due to loss of tocolytic effect of catecholamines leading to uterine hypertonus [24]. Our findings align with the catecholamine suppression hypothesis after CSE as fetal heart rate decreases in direct relation to intrathecal bupivacaine dose. Furthermore, hypotension may contribute to the fetal bradycardia. Although the phenylephrine dose was not statistically significant, it is significant when combined with CSE. This finding might be a surrogate outcome for the role of hypotension on the development of fetal bradycardia. Hence, it highlights the importance of preventative measures to avoid hypotension when using CSE technique for labor analgesia.

Our study demonstrates that the relationship between the evaluated predictors and the response variable is non-linear. PCR reduces the number of dimensions while keeping most of the information and variability contained in the predictors. Our study shows that most of the information is contained in the first two principal components; however, the minimum error is obtained with 13 principal components. This is because even directions with low eigenvalues contribute significantly to the model’s predictive value [25]. PCR is still a linear model that uses directions as the new predictors. When we compare the mean square error of PCR with that of ridge regression, some similarities come to our attention. Both statistical learning methods use the information contained in all the predictors without performing variable selection. The relatively large MSE exhibited by both PCR and ridge regression could be related to colinear variables, inclusion of non-contributory features, or non-linear relationships. To clarify the cause for the large errors, we fit an elastic net model that combines ridge regression with the lasso. This model adds a certain degree of variable selection. Elastic net performed even worse in terms of prediction accuracy. This finding highlights the fact that although the contribution of some features may be small, their contribution to prediction of a multifactorial response may still be important. We were then left with the possibility of having a non-linear relationship between predictors and response. A generalized additive model incorporating smoothing splines could not capture this non-linearity to its full extent. In contrast, the tree-based random forest model outperformed all other models as an accurate predictive tool.

Random forests have been used to classify abnormality of cardiotocograms in obstetrics with an accuracy of 93% [26]. Arif et al. identified tracing variability-related factors, accelerations, and uterine contractions as the most important variables in their random forest model. To our knowledge, our study is the first regression decision tree method used to predict changes in fetal heart rate after neuraxial analgesia. CSE and bupivacaine dose are among the most important variables identified by our random forest model, in agreement with the multiple regression findings. In addition, BMI and duration of stage 1 of labor were also at the top of variable importance to predict drops in fetal heart rate. The association between BMI and fetal heart rate may be due to technical factors such as the lack of reliability of the tracing due to body habitus [27]. On the other hand, maternal overweight and obesity affect the normal trajectory of fetal heart rate progression, which is amplified as pregnancy advances [28]. In addition, Liu et al. found that high BMI is associated with fetal bradycardia and adverse neonatal outcomes [29]. Finally, although no study has been published linking labor duration with fetal bradycardia, it is known that epidural analgesia prolongs the second stage of labor but not the first stage, whereas CSE may even shorten the first stage of labor. We may hypothesize that CSE and uterine effective contractions and hypertonus may coexist, and both predictors may be linked. Although this mechanism cannot be gleaned from our study, the coexistence of CSE and duration of stage 1 seem to work together to predict fetal heart changes in healthy pregnancies during labor.

Our study has several limitations. First, the retrospective nature of our analysis confers shortcomings to our study, such as information bias. Data entry into the institutional electronic information system occurs in real-time at a 5-minute interval. Therefore, some variables change and recover between measurements and might not be captured. Second, given the complexity and multifactorial influences on fetal heart rate, our models may fall short by disregarding important variables that may affect the response. However, most of the relevant and easily available variables were included and provided excellent predictive accuracy when taken together by our random forest model. The main strength of our study lies in the simultaneous evaluation of inference and prediction using different machine learning techniques. By comparing various models, we shed light on the nature of the relationships between predictors and response and on the variables implicated in these relationships. Also, we minimized the risk of overfitting by employing dataset splitting into training and test sets and using cross-validation. Our work revolved around optimizing the bias-variance trade-off for the different methods evaluated. Future research should focus on identifying additional variables that may be associated with the response so that our model can be further tuned up.

## Conclusion

In conclusion, use of CSE, presence of decelerations, total dose of bupivacaine, and total dose of vasopressors after CSE are associated with decreases in fetal heart rate in healthy parturients during labor. Prediction of changes in fetal heart rate can be approached with a tree-based random forest model with good accuracy with important variables that are key for the prediction, such as CSE, BMI, duration of stage 1 of labor, and dose of bupivacaine.

## Electronic supplementary material

Below is the link to the electronic supplementary material.

**Additional file 1**

## Data Availability

The datasets used and/or analyzed during the current study are available from the corresponding author on reasonable request.

## List of Abbreviations

ASA: American Society of Anesthesiologists
BMI: Body mass index
CSE: Combined spinal-epidural
GAM: General additive models
MSE: Mean square error
PCR: Principal components regression
PCA: Principal components analysis
OLS: Ordinary least squares
