# Supplementary material for: Fetal heart rate changes and labor neuraxial analgesia: a machine learning approach
Source: BMC Pregnancy Childbirth. 2023 May 22;23:329. doi: 10.1186/s12884-023-05632-3 (PMC10201770; doi:10.1186/s12884-023-05632-3)
Supplement: Supplementary file 1 — Additional file 1 [file 12884_2023_5632_MOESM1_ESM.docx]

**Fetal Heart Rate Changes and Labor Neuraxial Analgesia: A Machine Learning Approach**

**Supplement 1**

**Statistical Learning Methods: Background and Mathematical Rationale**

**Principal Components Regression**

Principal Components Regression (PCR) is a supervised learning method that serves the purpose of modeling the relationship between predictor variables and response. In contrast to linear regression, PCR uses the principal components (directions) as predictors [1]. With this method, the original predictors are transformed into a new set of orthogonal uncorrelated variables called principal components of the correlation matrix. This transformation ranks the new orthogonal components in order of their importance (as they contain most of the variance in the data) to effect variance reduction. After eliminating the least important components, a multiple regression of the response on the reduced set of principal components is fit using ordinary least squares (OLS). The calculated regression coefficient is then mathematically transformed into a set of coefficients corresponding to the original variables. These new coefficients are principal component estimators [2].

The usual least squares regression may be inappropriate in a case where the matrix of predictors is expected to be highly correlated or when the focus is on future prediction. The model


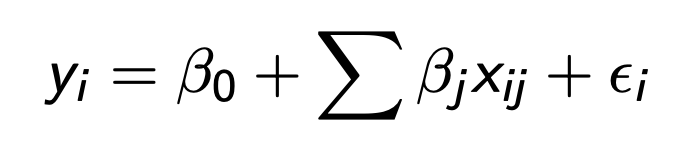


Is better represented by


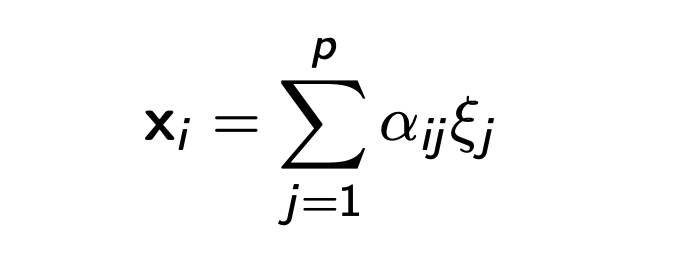


Where ξ_j_ represents the principal components of the predictor variables. Then, we can model


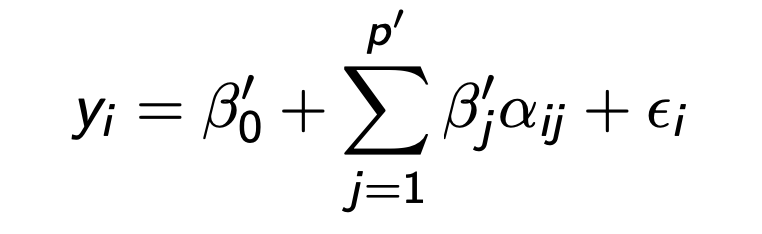


For some p` < p. The assumption is that most of the variation in the response occurs in the direction of large variations of the predictor variables (Figure 1).


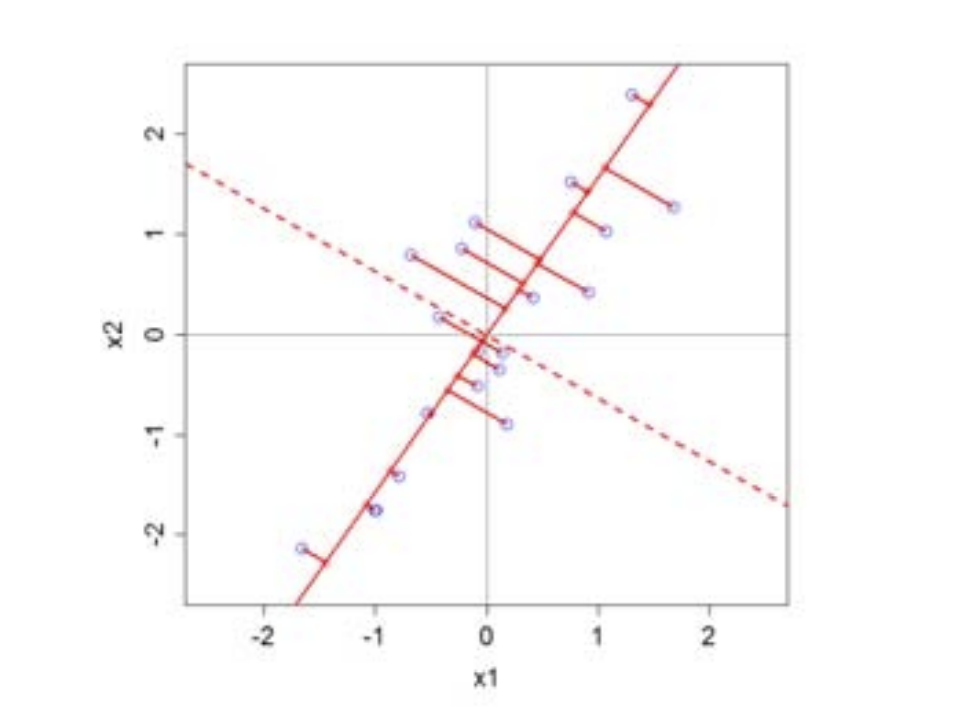


**Figure 1.** Maximum variance of predictor along a principal component

Using matrix notation, in OLS the regression coefficients are estimated using the formula


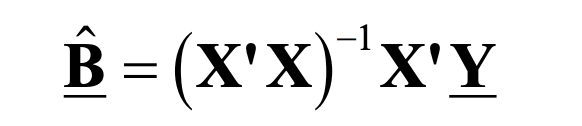


Since the variables are standardized, **X’X = R**, where **R** is the correlation matrix of independent variables. To perform PCR, the independent variables are transformed into principal components. Mathematically, this can be written as:


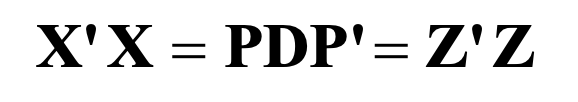


Where **D** is a diagonal matrix of eigenvalues of **X’X, P** is the eigenvector matrix of **X’X**, and **Z** is the matrix of principal components. By **P** being orthogonal, **P’P = I**. The principal components are linear combinations of the predictors. When eigenvalues are small, we assume that multicollinearity exists, and that principal component can be omitted. Then, the results can be transformed back to the **X** scales to obtain the estimated coefficients **B**. Although the estimates have added bias, we expect this effect to be more than offset by the decrease in variance. Mathematically, the estimation formula is:


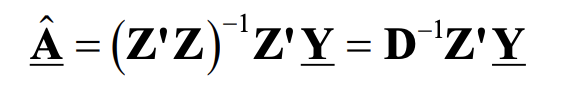


The two sets of regression coefficient are related as follows:


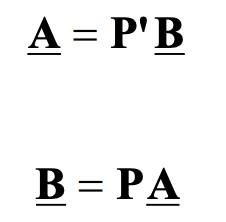


**Random Forest**

Random forest is an ensemble statistical learning method that can be used for regression and classification problems. It breaks the feature space into small fractions to grow a randomized tree predictor on each piece of data, and then aggregates those predictors together [3]. The method has gained popularity because of the simplicity of use without the need to tune parameters, and the ability to handle high-dimensional spaces.

A random forest is a predictor consisting of M randomized regression trees [4]. The trees are built from bootstrap training samples. Every time a split on a tree happens, a random sample of m predictors out of p total predictors is used as splitting candidates. We obtain the prediction for a new observation with known predictors by averaging the predictions across the decision trees obtained by bagging with limited number of predictors per split as shown by the formula below.


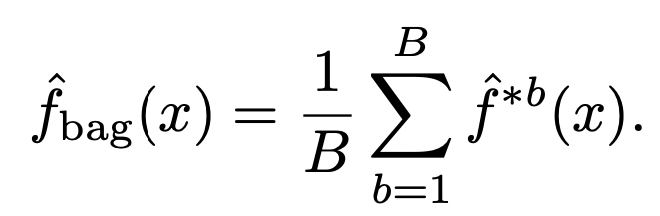


Where f-hat(x) is the predicted response, B is the number of decision trees, and f-hat^*b^(x) is the predicted response of each tree. Since random forests combines multiple decision trees, the interpretation is more difficult compared to a single tree. However, the relative importance of each predictor can be obtained by calculating the total amount that the residual sum of squares (RSS) is decreased over a given predictor over B number of trees. The larger the RSS reduction over the predictor, the more important such predictor is for predicting the response.

**Ridge Regression**

Linear models are easy to interpret and simple to understand; however, depending on sample size and distribution of data, they may be associated with overfitting. In order to mitigate this risk, different methodologies aim at reducing variance without a concomitant increase in bias to improve model prediction accuracy. One of those techniques is ridge regression. Ridge regression uses shrinkage of linear regression coefficients without getting to the point of selecting variables [5]. In many problems where data are not obtained from experimental design, non-orthogonality of predictor variables makes it impossible to assign proper weight to the individual features, thereby limiting the predictive accuracy of a linear model.

Ridge regression employs a penalty function governed by a tuning parameter that affects the loss function of residual sum of squares of linear regression. As the tuning parameter increases, the model becomes less flexible leading to lower variance and higher bias. The penalty function has the tuning parameter and the estimate of the coefficients β_j_ that minimizes the following function:


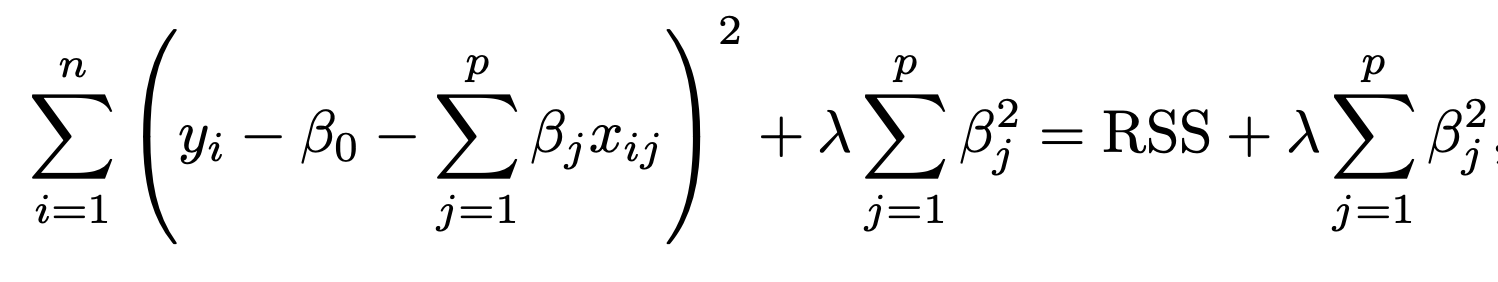


Where the tuning parameter λ is a non-negative number that is determined by cross-validation. The term λΣ_j_β_j_^2^ is the penalty function that can shrink the coefficient estimates to approach 0 (never equal to 0). The square of the coefficient estimates represents the L2 norm (distance from the zero coordinates). If we analyze the contours for the beta coefficient estimates, we see that the ridge regression represents the first point at which the coefficient contour intersects with the constraint imposed by the penalty function. Since the constraint of ridge regression is circular (quadratic), this intersection does not occur at the axes. As a result, the coefficients shrink but are never equal to zero (Figure 2).


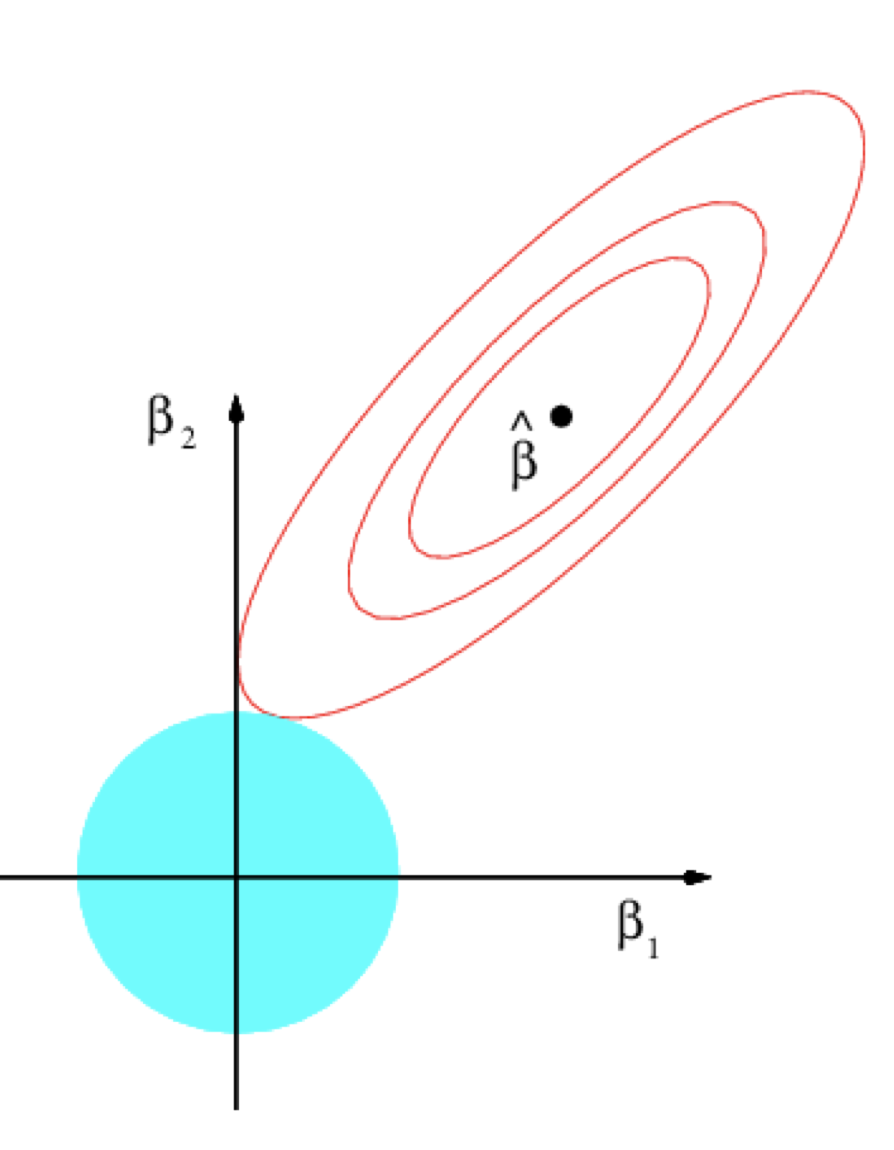


**Figure 2.** Contours of the errors for the coefficients and constraint function for ridge regression. The solid blue area is the constraint region. The ellipses are the contours of residual square sums

**General Additive Models**

General additive models (GAM) extend the linear regression models to include non-linear relationships between some or all features and the response.


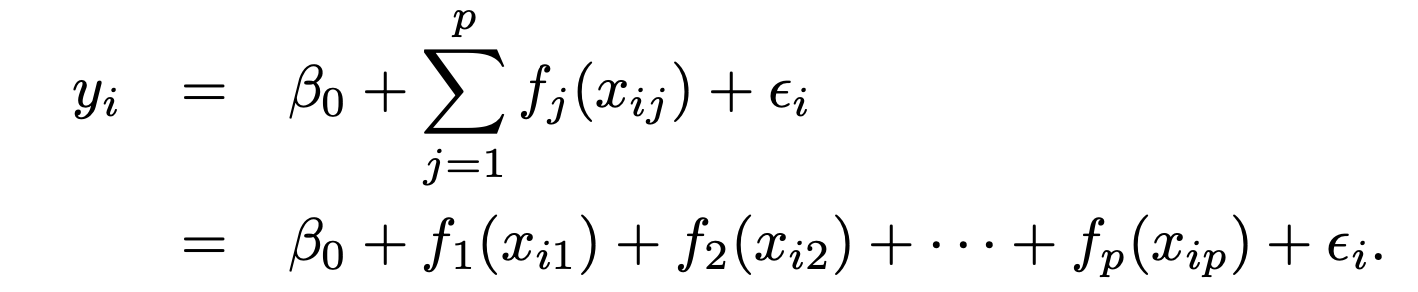


Where f_j_(x_ij_) represents a non-linear function that is different for each feature [6]. Although non-linearity is part of the function, the interpretation is still part of the linear framework. The functions f_j_(x_ij_) are nonparametric in the sense that the shape of predictor functions is determined by data directly and not by a small set of parameters. This allows for prediction without knowing the patterns beforehand. The f_j_ function can be expressed in terms of β (weights) and a basis expansion b:


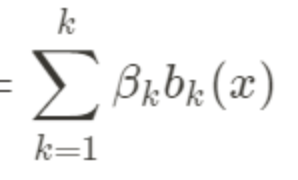


The basis expansion introduces non-linearity into each predictor-response relationship and corresponds to the general term of spline. Splines are smooth functions that can be understood as polynomials that cover a small range. The number of splines is a parameter that needs to be defined when this model is used. The parameter λ penalizes the splines. As λ value increases, the spline gets smoother until it becomes a straight line. The optimal value for λ is determined by cross-validation.

**Elastic Net**

Elastic net regression is a regularization method that uses the penalization of lasso and ridge regression on the loss function of OLS regression [7,8]. Elastic net improves the lasso limitations. It incorporates a quadratic component to the penalty function (the ridge regression component) to make the lasso more convex (Figure 3).


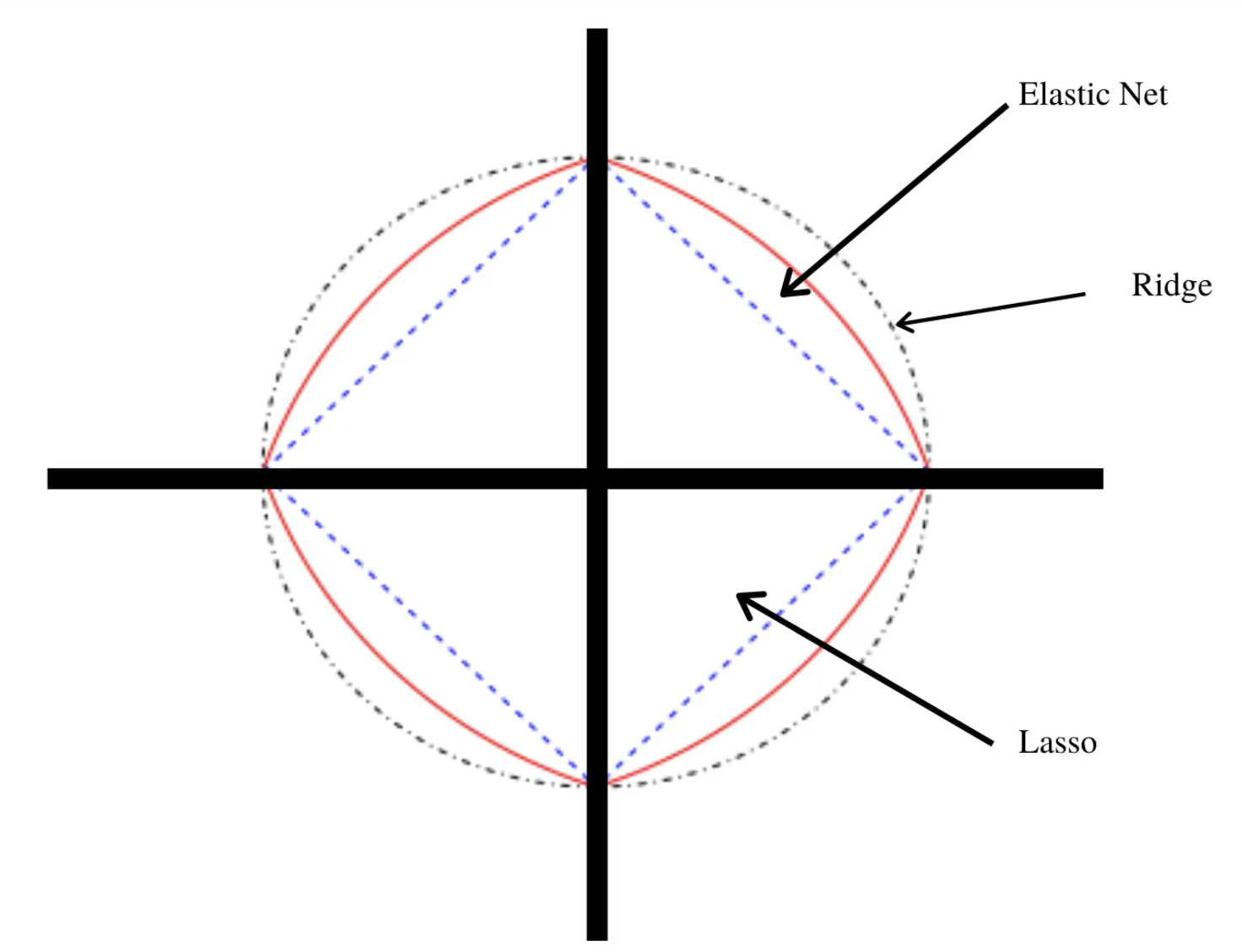


**Figure 3.** Constraints for the lasso, ridge regression and elastic net. Elastic net is intermediate constraint between the other two methods. Note the singularity at the vertices for elastic net.

The procedure to find the elastic net coefficient estimates starts with calculating the ridge regression coefficients followed by shrinkage of those coefficients using the lasso algorithm. Figure 3 displays the constraint area for the elastic net compared to the lasso and ridge regression. In the figure, singularity at the vertices is observed. This singularity is necessary for variable selection. On the other hand, the double shrinkage leads to low prediction efficiency and high bias. To overcome this problem, the coefficients are rescaled by multiplying them by (1+λ_2_). Convexity also depends on the correlation-dependent grouping of the selected variables. At high levels of correlation between variables, convexity causes a greater grouping and a larger number of variables included in the sample. Elastic net performs well when there are highly correlated independent variables. During the shrinkage procedure, the L1 norm of the lasso selects variables. On the other hand, the L2 norm of ridge regression makes the L1 component of the penalty more stable.

Elastic net aims at minimizing the following expression:


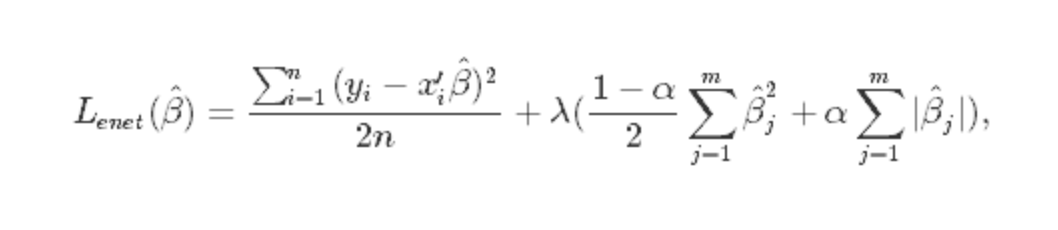


Where α is the parameter that fluctuates between ridge regression (α=0) and lasso (α=1). This means that the two parameters require tuning: α and λ. This tuning can be done with cross-validation.

**References**

1. Draper NR, Smith H. Applied regression analysis. John Wiley & Sons; 1998 Apr 23.
2. Gunst RF. Regresion analysis with multicollinear predictor variables: definition, derection, and effects. Communications in Statistics-Theory and Methods. 1983 Jan 1;12(19):2217-60.
3. Breiman L. Random forests. Machine learning. 2001 Oct;45(1):5-32.
4. Biau G, Scornet E. A random forest guided tour. Test. 2016 Jun;25(2):197-227.
5. McDonald GC. Ridge regression. Wiley Interdisciplinary Reviews: Computational Statistics. 2009 Jul;1(1):93-100.
6. Liu H. Generalized additive model. Department of Mathematics and Statistics University of Minnesota Duluth: Duluth, MN, USA. 2008 Dec;55812.
7. Ogutu JO, Schulz-Streeck T, Piepho HP. Genomic selection using regularized linear regression models: ridge regression, lasso, elastic net and their extensions. InBMC proceedings 2012 Dec (Vol. 6, No. 2, pp. 1-6). BioMed Central.
8. Zou H, Hastie T. Regularization and variable selection via the elastic net. Journal of the royal statistical society: series B (statistical methodology). 2005 Apr;67(2):301-20.
